# Stroke Prevention Therapy and Prevalence of Risk Factors Among Patients With Atrial Fibrillation at King Fahad University Hospital in Al Khobar: A Retrospective, Single-Center Study

**DOI:** 10.7759/cureus.12493

**Published:** 2021-01-05

**Authors:** Maryam A Alalwan, Fatimah Al-Ohaid, Huda M Alhajjaj, Ahlam Al Hazeem, Ghadeer H AlJulaih, Rabab Al-Khedher, Abdullah Alshehri, Noor-Ahmed Jatoi

**Affiliations:** 1 Internal Medicine, Imam Abdulrahman Bin Faisal University, Al Khobar, SAU; 2 Internal Medicine, Imam Abdulrahman Bin Faisal University, King Fahad University Hospital, Al Khobar, SAU

**Keywords:** atrial fibrillation, anticoagulants, stroke, antiplatelets, prevention

## Abstract

Background

Atrial fibrillation is the most common cardiac arrhythmia in clinical practice. It represents a significant health impact as it is greatly associated with increased risk of mortality and morbidity, most importantly stroke and systemic thromboembolism.

Aim

This study aims to determine the risk factors of atrial fibrillation, to identify stroke and bleeding risk factors among patients with atrial fibrillation, to assess the trend of stroke prevention management and the influence of CHA2DS2-VASc and HAS-BLED scores on choosing the treatment.

Methods

This study was performed using all the medical records of 395 patients with Atrial fibrillation who were admitted between 2011-2019 at King Fahd University Hospital, Al-Khobar, Saudi Arabia. The review process included demographic data of the patients and the calculation of stroke and bleeding risk by CHA2DS2-VASc and HAS-BLED scores.

Results

The median age of the population was 72 years old. Hypertension was the most common risk factor for atrial fibrillation (78.2%), followed by diabetes mellitus (61.0%), dyslipidemia (60.0%), coronary artery disease (41.0%), myocardial infarction (18.7%), and congestive heart failure (29.4%). Regarding the management, (42.5%) of the patients were on a combination of both anticoagulants and antiplatelet therapy, while (33.2%) were on anticoagulant therapy only, (17.5%) were on antiplatelets only, and (5.8%) were not on medication. The increased use of anticoagulants and combined therapy was related to the percentage of a high-risk group of thromboembolic events reaching up to (34.5%) and (45.7%), respectively, which is statistically significant. Moreover, the prescription of warfarin declined in the last five years of our study, while the use of non-vitamin K antagonist oral anticoagulants increased.

Conclusion

Atrial fibrillation is more prevalent in females, hypertension was the most common risk factor for atrial fibrillation, followed by diabetes mellitus, and dyslipidemia. Most of the studied population was categorized as a high risk of stroke and bleeding according to CHA2DS2-VASc and HAS- BLED scores. The majority of the atrial fibrillation patient were taking anticoagulants and combined treatment as a stroke prevention therapy. Non-vitamin K antagonist oral anticoagulant prescription increased over warfarin in recent years.

## Introduction

Atrial fibrillation (AF) is the most common cardiac arrhythmia in clinical practice, which represents a significant health impact as it is greatly associated with increased mortality and morbidity, most importantly stroke [1]. It’s expected that by 2050, AF will affect over 50 million people around the world [2].

During a mean follow-up study of 3.2 years duration, on 10,654 South Korean AF patients, 1022 (9.6%) of them were diagnosed with ischemic stroke. The overall incidence rate of ischemic stroke was 30.8/1000 person-years [3].

AF occurs in a striking diversity of clinical settings, from patients without risk factors to those with advanced underlying heart disease. Multiple lines of evidence demonstrate a common genetic predisposition since the early 1930s, the first GWAS (genome-wide association study) that was done on an Icelandic population, 550 AF patients and 4476 controls in 2007, detected an association between variants at chromosome 4q25 and AF susceptibility, but the mechanism is still not understood [4].

It is well established that the increasing age of the population is associated with a higher prevalence of AF. Multiple studies have evaluated other factors that increase the risk of AF and the subsequent thromboembolic events associated with the disease. Major risk factors were comorbid diseases such as hypertension (HTN), diabetes mellitus (DM), obesity, dyslipidemia, obstructive sleep apnea, coronary artery disease (CAD), chronic kidney disease (CKD), smoking, and alcohol consumption [1,5]. Most of the risk factors are modifiable, thus identifying the risk factors of AF is crucial [6]. Prevention of stroke is the main aim of medical treatment in patients with AF this could be achieved by oral anticoagulants (OACs) [5]. Two main categories of anticoagulants can be classified as vitamin K antagonist (VKA) warfarin and non-vitamin K antagonist oral anticoagulants (NOACs; apixaban, dabigatran, edoxaban, and rivaroxaban). Regarding antiplatelets, the main agents that had been studied were aspirin and clopidogrel which were less effective than anticoagulants in the prevention of stroke in patients with atrial fibrillation with a similar risk of bleeding [7]. Warfarin has been the classic anticoagulant, in spite of its well-known side effects and compliance challenges for patients. A false conception about warfarin is that in case of bleeding, it can rapidly be reversed, while the majority of the trials reported that warfarin reversal needs 24 hours to halve the INR value, and NOACs have both a quick onset of action and a brief biological half-life [8].

Moreover, NOACs are more cost-effective than Warfarin, as demonstrated by a study conducted in 2019 studying the cost-effectiveness of apixaban for stroke prevention in non-valvular atrial fibrillation in Saudi Arabia [9]. Since NOACs were released in the market they have been increasingly used to prevent stroke in atrial fibrillation patients, an observational study was conducted by (Boriani et al. in 2018) to assess prevention strategies in 27 European countries, concluded that between 2013-2016 the patients who were on OACs therapy were much more than those who were on antiplatelet therapy and that NOAC accounted for 40.9% of OACs. The preference factors of using OACs were age, HTN, old ischemic stroke, symptomatic AF, and planned cardioversion or ablation [10]. A recent study published in 2020 illustrated that the prescription of OACs has markedly raised from 42.9% in 2014 to 72.7% in 2018 [11].

The stroke risk assessment must be done prior to the establishment of medical treatment. It is strongly recommended to be assessed using CHADS2 and CHA2DS2-VASc scores [12]. The main risk assessment tool is CHADS2, updated with the more recent CHA2DS2-VASc to obtain a more accurate evaluation of low-risk patients [8]. On the other hand, the variation of the bleeding risk should be considered and assessed by the HAS-BLED score, which also aids in the decision making for the appropriate antithrombotic therapy [7].

This study is launched due to the lack of evidence-based studies that cover AF disease, its risk factors, and related complications in Al-Khobar. The purpose of the study is to determine the risk factors of atrial fibrillation, to identify stroke and bleeding risk factors among patients with atrial fibrillation, to assess the trend of stroke prevention management in AF patients and the influence of CHA2DS2-VASc, European heart association score (EHRA) and HAS-BLED scores on choosing the appropriate treatment at King Fahd University Hospital (KFUH), Al-Khobar.

## Materials and methods

This observational retrospective single-center study was conducted over a period of eight months, from September 2019 to April 2020. It included 395 patients with AF that were admitted between 2011-2019 at KFUH, Al Khobar, Saudi Arabia.

Subjects

Inclusion criteria include all 395 patients with AF who were admitted between 2011-2019 at KFUH in Al Khobar 2011- 2019. Exclusion criteria include none (all 395 patients with AF were included in the study to avoid bias).

Sample size and sampling technique

The data of the 395 patients were extracted from a computer-generated database of the patients' medical records (QuadraMed) in KFUH with an acceptable data completion rate of >98%. A patient was considered to have an AF when AF was documented in the record provided by the QuadraMed. We included only one hospital admission per patient and excluded any second hospital admission if produced.

The data collection sheet was designed by the study authors. It contained the patient's demographic data, risk factors of atrial fibrillation and thromboembolic events including, CAD, DM, HTN, congestive heart failure (CHF), dyslipidemia, CKD, and adverse lifestyle (smoking and obesity). In addition to CHA2DS2-VASc and HAS-BLED scores, laboratory values of lipid profile, kidney function test, and the pharmacological management the patient's receiving for stroke prevention.

The calculation of the CHA2DS2-VASc score is accomplished by adding (1) or (2) points for each of the following risk factors: CHF (1), HTN (1), age ≥75 years (2), DM (1), stroke, transient ischemic attack (TIA) or thromboembolism (2), vascular disease: myocardial infarction (MI), peripheral vascular disease or aortic atherosclerosis (1), age 65-74 years (1), and female Sex (1). According to American Stroke Association ASA and American Heart Association (AHA) guidelines patients are classified into low (score is 0), intermediate (score is 1) and high (score ≥2) risk group for stroke [7].

The calculation of HAS-BLED score is achieved by adding (1) point for each of the following risk factors: HTN, abnormal renal function, abnormal liver function, stroke, bleeding tendency or predisposition, labile international normalized ratio (INR), elderly (age >64 years), drugs (nonsteroidal anti-inflammatory or antiplatelet) and alcohol use [7]. According to the American College of Chest Physicians guidelines (2012), patients are categorized based on their HAS-BLED score into low (score is 0), moderate (score is 1-2), and high (score ≥3) risk for bleeding [13].

The information obtained from these records will be used for research purposes only and patients’ identities will not be revealed to keep the patients’ confidentiality.

The institutional review board (IRB) approval from Imam Abdulrahman bin Faisal University, office of the vice president for research & higher studies was obtained before launching this study (IRB # -UGS-2019-01-326, approval date 21/9/2019).

Data analysis

Data were tabulated using a Microsoft Excel sheet and coded for analysis using Statistical Package For The Social Sciences (SPSS) 23.0 (IBM Corp., Armonk, NY) with a 95% confidence interval. Data were presented as mean, median, and standard deviation (SD), and a P <0.05 was considered as statistically significant. Regarding the age, median and quartile were used instead of the mean, because the age of our sample was not normally distributed (Non-parametric). Different statistical tests were also used in the analysis, such as the independent samples t-test for comparison between continuous variables and chi-square test for comparison between categorical variables. In addition, Fisher's exact test was used to set the association between categorical variables, as well as a one-way ANOVA test to determine the statistical significance between independent variables.

## Results

Clinical characteristics and risk factors of AF patients

Most patients were Saudi 296 (74.9%) compared to non-Saudi 99 (25.1%). The median age of the study sample was 72 years old. The age distributions are shown in Table 1, most of the patients 168 (42.5%) were elderly ˃ 75, 99 (25.1%) were between 65 and 75, while the rest of the patients 128 (32.4%) were ≤ 65 years old.

**Table 1:** Clinical characteristic and risk factors of AF in the population, total (n=395). CAD= coronary artery disease, MI= myocardial infraction, DM= diabetes mellitus, HTN= hypertension, CHF= congestive heart failure, CKD= chronic kidney disease, TIA= transient ischemic attack, AF= atrial fibrillation

Sample characteristic	Frequency	Percent (%)
Saudi	296	74.9
Non-Saudi	99	25.1
Male	179	45.3
Female	216	54.7
Age˃ 75 y	168	42.5
Age˃ 65 y and ≤ 75 y	99	25.1
Age≤ 65	128	32.4
CAD	162	41.0
MI	74	18.7
DM	241	61.0
Smoking	22	5.6
HTN	309	78.2
CHF	116	29.4
Dyslipidemia	237	60.0
CKD	79	20.0
TIA	24	6.1
stroke	90	22.8
Median age: 72 years old Percentile 25: 61 years old 50: 72 years old 75: 81 years old		

In addition, HTN was the most common risk factor for AF 309 (78.2%), followed by DM 241 (61.0%), and dyslipidemia 237 (60.0%). Other less common risk factors were CAD 162 (41.0%), MI 74 (18.7%), CHF 116 (29.4%), CKD 79 (20.0%), TIA 24 (6.1%) and stroke 90 (22.8%).

Thromboembolism prevention therapy in the population

These patients were on anticoagulant and antiplatelet therapy as prevention of stroke. Among these patients there were 23 (5.8%) who were not taking any medications, 131 (33.2%) were only on anticoagulant therapy which includes warfarin, heparin, and NOAC, 69 (17.5%) were only on antiplatelet therapy which includes aspirin and clopidogrel, 168 (42.5%) of them were on a combination of both anticoagulant and antiplatelet therapy and (1%) could not be assessed due to missing data from the electronic records as shown in Table 2.

**Table 2:** Prevalence of the usage of medications as prevention of stroke among the patients’ sample.

Medications	Frequency	Percentage (%)
Off medication	23	5.8
Anticoagulants	131	33.2
Antiplatelets	69	17.5
Both anticoagulants and antiplatelets	168	42.5
Missing	4	1

In the study sample, most of the patients 218 (55.2%) were taking Aspirin as antiplatelet management while 86 (21.8%) were taking clopidogrel. There were no major differences in heparin 136 (34.4%) and Warfarin 124 (31.4%) management among the patients. NOAC medication use was rising over the years and 104 (26.3%) of the sample were taking NOAC.

**Figure 1:**
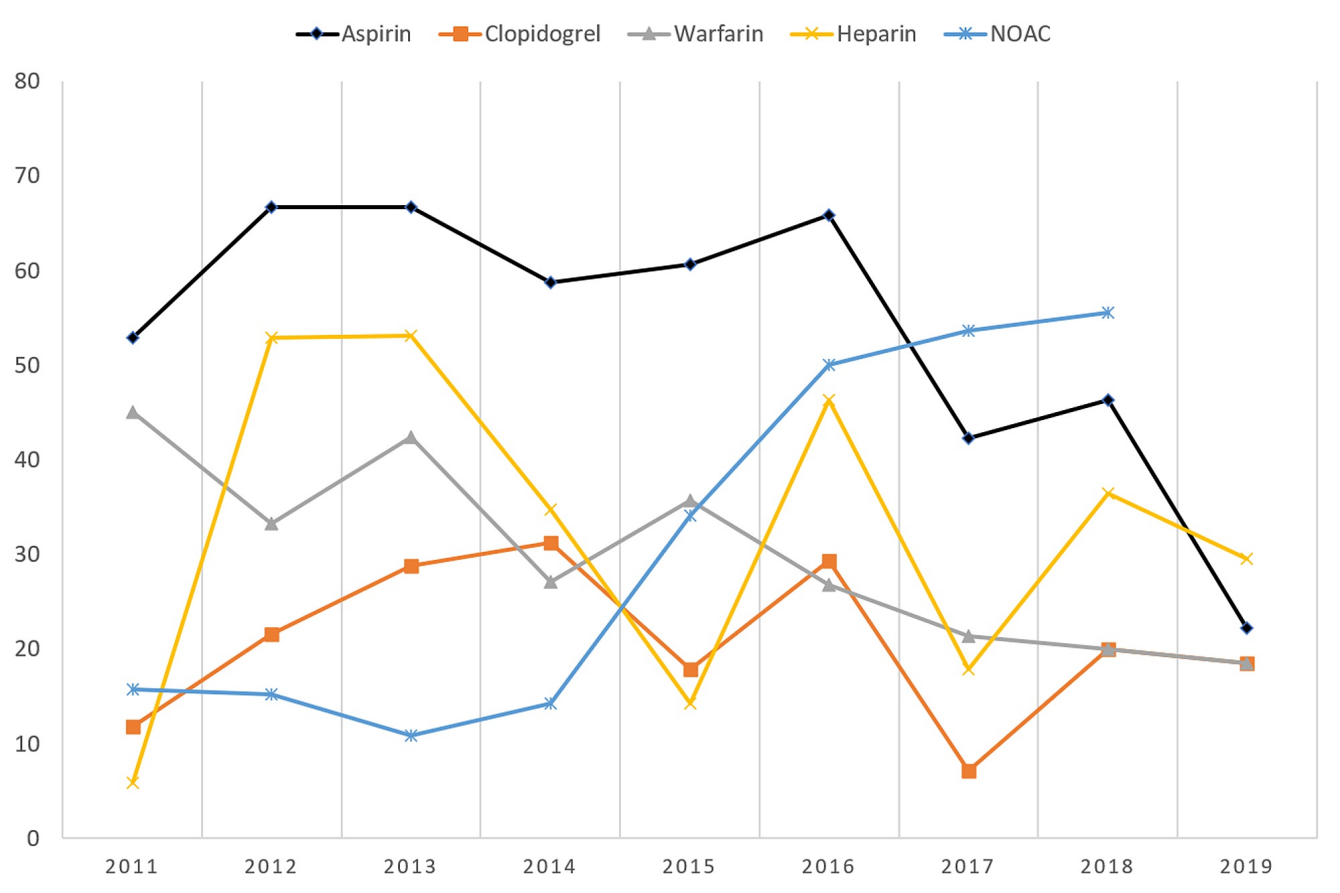
Prevalence (%) of prescription of medication in relation to year. NOAC: non-vitamin K antagonist oral anticoagulants

Table 3 and Figure 1 show the prevalence of medication prescription in relation to years. The prescription of NOAC medications has been increasing from 2011 6 (11.8%) to 2019 15 (55.6%), where more than half of the patients with AF were on NOAC. There is also a decrease in the prescription of warfarin from 2011 to 2019, 23 (45.1%) to 5 (18.5%), respectively.

**Table 3:** Prevalence of prescription medication in relation to year. NOAC: non-vitamin K antagonist oral anticoagulants

Year	Aspirin (%)	Clopidogrel (%)	Warfarin (%)	Heparin (%)	NOAC (%)
2011	27 (52.9)	6 (11.8)	23 (45.1)	3 (5.9)	6 (11.8)
2012	34 (66.7)	11 (21.6)	17 (33.3)	27 (52.9)	8 (15.7)
2013	44 (66.7)	19 (28.8)	28 (42.4)	34 (51.5)	10 (15.2)
2014	27 (56.2)	15 (31.2)	13 (27.1)	16 (33.3)	5 (10.4)
2015	17 (60.7)	5 (17.9)	10 (35.7)	4 (14.3)	4 (14.3)
2016	27 (65.9)	12 (29.3)	11 (26.8)	19 (46.3)	14 (34.1)
2017	11 (39.3)	2 (7.1)	6 (21.4)	5 (17.9)	13 (46.4)
2018	25 (45.5)	11 (20.0)	11 (20.0)	20 (36.4)	29 (52.7)
2019	6 (22.2)	5 (18.5)	5 (18.5)	8 (29.6)	15 (55.6)

Table 4 shows the different treatment modalities in relation to different risk factors and compares the clinical characteristics and the prevention therapy. Patients were divided into four groups according to the prevention therapy they received: the off medications group, antiplatelet medications group, anticoagulant medications group, and lastly those who were on both antiplatelets and anticoagulants. 

**Table 4:** Relation between the patient’s risk factors for AF and the management. CAD= coronary artery disease, MI= myocardial infarction, DM= diabetes mellitus, HTN= hypertension, CHF= congestive heart failure, CKD= chronic kidney disease, TIA= transient ischemic attack, AF= atrial fibrillation

Sample risk factors	Off medications (%)	Anticoagulants (%)	Antiplatelets (%)	Combination of anticoagulants and antiplatelets (%)	P-value
Age ≤ 65 y	9 (7.1)	54 (42.5)	25 (19.7)	39 (30.7)	0.00
Age ˃ 65 y	14 (5.3)	77 (29.2)	44 (16.7)	129 (48.9)	
Age ≤ 75 y	17 (7.5)	81 (35.8)	44 (19.5)	84 (37.2)	0.03
Age ˃ 75 y	6 (3.6)	50 (30.3)	25 (15.2)	84 (50.9)	
Saudi	18 (6.1)	97 (33.1)	51 (17.4)	127 (43.3)	0.96
Non-Saudi	5 (5.1)	34 (34.7)	18 (18.4)	41 (41.8)	
CAD	7 (4.3)	29 (17.9)	34 (21.0)	92 (56.8)	0.00
MI	3 (4.1)	9 (12.2)	13 (17.6)	49 (66.2)	0.00
DM	10 (4.2)	84 (35.0)	36 (15.0)	110 (45.8)	0.08
HTN	11 (3.6)	100 (32.5)	49 (15.9)	148 (48.1)	0.00
CHF	2 (1.7)	38 (32.8)	18 (15.5)	58 (50.0)	0.07
Dyslipidemia	4 (1.7)	65 (27.4)	39 (16.5)	129 (54.4)	0.00
CKD	9 (11.4)	16 (20.3)	18 (22.8)	36 (45.6)	0.00
TIA	1 (4.2)	11 (45.8)	2 (8.3)	10 (41.7)	0.44
Stroke	2 (2.2)	23 (25.6)	7 (7.8)	58 (64.4)	0.00

Among the risk factors; age, CAD, MI, HTN, dyslipidemia, CKD, and stroke were statistically significant. The majority of the HTN patients were on anticoagulants 100 (32.5%) followed by antiplatelets 49 (15.9%). Regarding the patients with age > 65, the use of anticoagulants was higher than antiplatelets 77 (29.2%) and 44 (16.7%) respectively. For patients who had a stroke, most of them were on combination therapy 58 (64.4%), followed by anticoagulants 23 (25.6%), antiplatelets 7 (7.8%), and 2 (2.2%) of them were not on any medications.

Comparison of CHA2DS2-VASc and HAS-BLED score

The risk of stroke in patients with AF was measured using the CHA2DS2-VASc score. Most of the study sample were categorized as high risk for stroke 339 (85.8%), compared with 26 (6.6%) intermediate-risk and 21 (5.3%) low risk. 

There were 9 (2.3%) of the patients who could not be scored due to the missing information from the hospital system. The mean of CHA2DS2-VASc was 4.1 ±SD 2.1 as shown in Table 5. 

**Table 5:** CHA2DS2-VASc and HAS-BLED risk categories in the sample.

Category		Frequency	Percentage %	Mean ±standard deviation
CHA2DS2-VASc risk score	Low risk (= 0)	21	5.3	4.1 ± 2.1
	Intermediate risk (= 1)	26	6.6	
	High risk (≥ 2)	339	85.8	
	Missing	9	2.3	
HAS-BLED risk score	Low risk (= 0)	16	4.1	2.6 ± 1.2
	Intermediate risk (= 1-2)	157	39.7	
	High risk (≥ 3)	212	53.7	
	Missing	10	2.5	

The tendency of bleeding among the sample was measured using HAS-BLED score as shown in Table 5. Most of the patients had a high risk of bleeding 212 (53.7%), 157 (39.7%) of them had an intermediate risk, while only a few of them 16 (4.1%) had a low risk. The mean was 2.6 ±SD 1.2. There were 10 (2.5%) of the patients who could not be scored due to the missing information from the hospital system.

**Figure 2:**
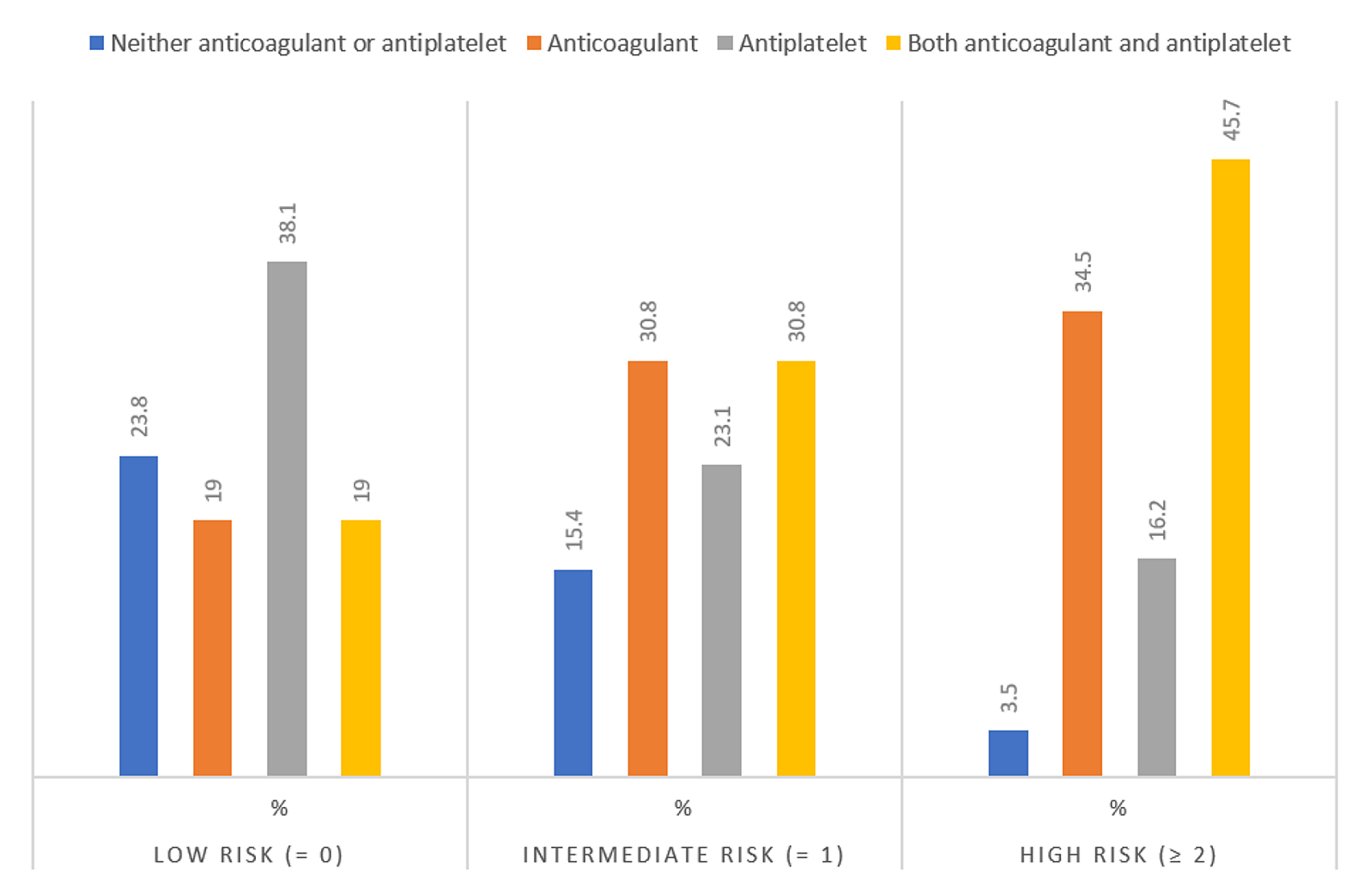
CHA2DS2-VASc risk categories in the sample.

Table 6 and Figure 2 show the trend on prescribing anticoagulants and antiplatelets in relation to the CHA2DS2-VASc score. The use of antiplatelets was more than anticoagulants in low-risk patients, 8 (38.1%) compared with 4 (19%), respectively. In contrast, the use of anticoagulant therapy was noted to be high in the high-risk group when the CHA2DS2-VASc ≥ 2 reaching up to 117 (34.5%), which is statistically significant (P = 0.00).

**Table 6:** CHA2DS2-VASc and HAS-BLED risk categories in the sample.

Category		Neither anticoagulants nor antiplatelets (%)	Anticoagulants (%)	Antiplatelets (%)	Both anticoagulants and antiplatelets (%)	Total (%)
CHA2DS2-VASc risk score	Low risk (= 0)	5 (23.8)	4 (19.0)	8 (38.1)	4 (19.0)	21 (5.4)
	Intermediate risk (= 1)	4 (15.4)	8 (30.8)	6 (23.1)	8 (30.8)	26 (6.7)
	High risk (≥ 2)	12 (3.5)	117 (34.5)	55 (16.2)	155 (45.7)	339 (87.8)
HAS-BLED risk score	Low risk (= 0)	5 (31.2)	7 (43.8)	2 (12.5)	2 (12.5)	16 (4.2)
	Intermediate risk (= 1-2)	6 (3.8)	70 (44.6)	32 (20.4)	49 (31.2)	157 (40.8)
	High risk (≥ 3)	10 (4.7)	52 (24.5)	35 (16.5)	115 (54.2)	212 (55.1)
P. Value = 0.00						

**Figure 3:**
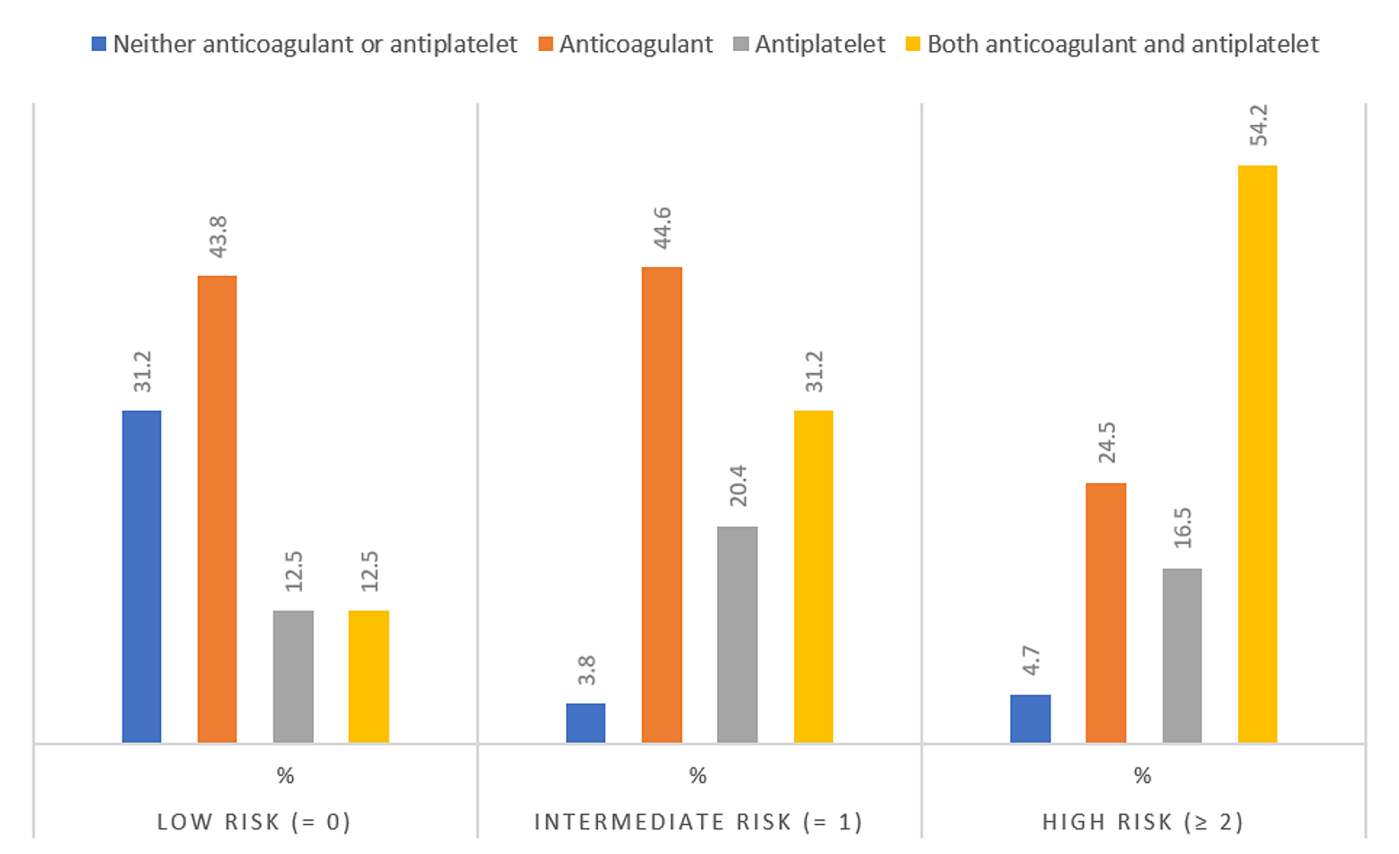
HAS-BLED risk categories in the sample.

Table 6 and Figure 3 show the trend on prescribing anticoagulants and antiplatelets in relation to the HAS-BLED score. The prescription of anticoagulants was higher in the intermediates group with HAS-BLED score (1-2) 70 (44.6%), while in the group with a high risk of bleeding HAS-BLED score (≥3) its percentage become 52 (24.5%). 115 (54.2%) of patients in high-risk group take a combination of both anticoagulants and antiplatelets (P = 0.00).

## Discussion

This observational retrospective study was conducted at KFUH in Al Khobar, Saudi Arabia. The study involved 395 patients who have AF, results have shown that the median age of our study's population is 72 years old which is corresponding with the common age of developing AF that is identified in the literature. It is well established that elderly patients with AF are nearly at five-folds risk of stroke than other patients with AF, but the exact mechanism is still not well-understood because elderly patients usually present with multiple comorbidities, therefore it is difficult to distinguish between the effect of comorbidities and age as a risk factor [14]. Some studies have hypothesized that AF could be attributed to age-related electrophysiological and structural changes of atrial myocardium. 

The percentage of females 54.7% was more than the percentage of males 45.3%, this result is consistent with a recent study conducted at King Abdulaziz University Hospital (KAUH), Jeddah, Saudi Arabia in 2019 to study the risk factors, etiologies, comorbidities, and outcomes of AF in Saudi Arabia. It suggested that, after adjustment for other risk factors, the risk of stroke in female patients with AF was two- to 4.5-fold greater than in male patients with AF [6]. Moreover, According to the CHA2DS2-VASc score our study's population is at increased risk of stroke since most of the population were elderly and females [15]. Upon reviewing the risk factors of AF in KFUH, HTN was found to be the most common risk factor 78.2% followed by DM 61.0%, dyslipidemia 60.0%, and CAD 41.0%. On the other hand, MI 18.7%, CHF 29.4%, CKD 20.0%, TIA 6.1%, and stroke 22.8% were less common. These results are in concordance with the results of the KAUH study, it indicated that HTN was the most common risk factor 73.1%, followed by valvular disease 58.7% and DM 53.3%, Obstructive sleep apnea 2.4%, and rheumatoid arthritis 3.0% were the least common comorbidities [6]. Similarly, a cross-sectional study was conducted at King Abdulaziz Medical City (KAMC) in Riyadh, Saudi Arabia in 2016. It found that HTN 79.5% was the most prevalent risk factor for stroke followed by DM 54.5%, female sex 54.5%, and age ≥ 65 years old 38.6% [13]. This is supported by another study that was done in 2011, including 18 centers in Saudi Arabia with a total of 400 patients. It showed that the most common comorbidities in patients with AF were: HTN 62.2%, DM 43%, dyslipidemia 44%, body mass index >25 77.7%, smoking 23% and heart failure 31.7% [16]. However, in our study, we could not detect the accurate percentage of the patients who had BMI > 25 and who were smokers due to the lack of this information in the patients' electronic medical records (QuadraMed) system in KFUH. Regarding HTN, it is the most common risk factor of AF which is reasonable, considering its possible effect on the heart muscles, blood vessels, and cardiac conduction system which may result in the development of arrhythmia [17]. 

Based on the current study results, it is well recognized that most of our study population were found to have a high prevalence of modifiable risk factors for thromboembolic events, HTN and DM were on top of them. Therefore, targeting these modifiable risk factors in lifestyle modification and health promotion programs must be reinforced in our clinical practice.

The prevalence of antiplatelets prescription was significantly lower than anticoagulants’, 17.5% and 33.2% respectively. This could be due to the superiority of anticoagulants in stroke prevention, as demonstrated in a study that was conducted in 580 centers in 33 countries (2009) on 7554 patients with AF to determine the effect of antiplatelet therapy on stroke prevention [18]. Adding to that, the majority of our population was categorized as high risk according to a CHA2D2S-VASc score of 85.8% which explains the increased use of anticoagulants in our population. Comparing to our finding of 85.8%, the percentage of patients who were at high risk of stroke was higher in a study reported by Al-Turaiki et al 92% upon assessing CHA2DS2-VASc score. This can be attributed to the high prevalence of modifiable risk factors as reported in their study. In contrast to the current study sample, the high-risk category of HAS-BLED score was more prevalent 53.7% in comparison to another study in which 27.7% of patients fell in a high-risk category, this may be due to their small sample size in which only 264 were eligible to be enrolled in their study [13]. 

In the current study sample, it is found that warfarin use declined in the last five years, while the use of NOAC increased, this can be attributed to NOAC's therapeutic features that favor their use over warfarin, which include: lower bleeding risk, less drug, and food interactions, unnecessity of frequent monitoring and wider therapeutic range [19,20]. In agreement with our results, a cross-sectional study on 112,187 patients with nonvalvular AF, was conducted to evaluate the trend on the prescription of OACs between the period of 2010-2017. It found that NOACs prescription has increased substantially since their introduction. 7,502 patients were started on OACs in the first quarter of 2017, of whom 5919 (78.9%) used NOACs and 1583 (21.1%) were on warfarin [21]. 

American College of Cardiology (ACC) and American Heart Association (AHA) recommended the use of NOAC over warfarin in NOAC eligible patients for preventing stroke and thromboembolic events. Adding to that, NOACs are found to be associated with lower bleeding risk excluding cases of moderate to severe mitral stenosis or mechanical heart valve, in which warfarin is recommended. Moreover, specific NOACs such as apixaban may have lower bleeding risk including intracranial hemorrhage in comparison to warfarin. Other NOACs particularly dabigatran and rivaroxaban are associated with less risk of Intra-cranial hemorrhage and renal adverse effects than warfarin in patients with AF over time [22,23]. 

In the current study, results showed that the majority 45.7% of patients who fell in the high-risk category of CHA2D2S-VASc score were prescribed a combined treatment of both anticoagulants and antiplatelets. Although prescription of combined therapy in the absence of indications (for example, coronary artery disease, cerebrovascular disease, and peripheral artery disease) still remains a common clinical practice to be used, there is limited evidence to support the use of combination therapy for stroke prevention only in patients with AF [23]. In addition to that 34.5% of those who were categorized as high-risk of stroke were prescribed anticoagulants, which is consistent with the AHA guidelines [15]. However, 6.2% of those who were at high risk for stroke were only on antiplatelet therapy. Similarly, of participants who were categorized as high-risk for stroke, only 3.5% received neither antiplatelets nor anticoagulants, this can be further explained by the fact that most of those patients have a high to moderate risk of bleeding according to HAS-BLED score. Despite that, it is found that the HAS-BLED score's impact on the treatment was not as significant as the CHA2D2S-VASc score's. 54.2% of the patients who were classified as high-risk according to HAS-BLED score have prescribed both anticoagulants and antiplatelets. This is consistent with the recommendation of the Royal College of Physicians of Edinburgh, which stated that HAS-BLED can aid in the identification of modifiable bleeding risks, but it should not be used alone to exclude patients from the use of Anticoagulant therapy [24].

Limitations

The diagnosis coding system in KFUH only includes inpatients therefore, the study sample is relatively small as it only included patients who had been admitted to the hospital from 2011 to 2019. The EHRA score which places the patients into four categories according to the severity of their symptoms couldn’t be assessed because many patients with AF were already diagnosed and there was no information documented in the patient’s file. Smoking and body mass index (BMI) of the patients were not assessed due to the lack of this information in the hospital system.

## Conclusions

We conclude that AF is more prevalent among females. Hypertension, diabetes mellitus, and dyslipidemia were the most common risk factors associated with AF in Saudi Arabia. Most of the studied population was categorized as a high risk of stroke and bleeding according to CHA2DS2-VASc and HAS-BLED scores. Thus, the majority of patients with AF were antithrombotic. Only 34.5 % of patients with AF who fell in the high-risk category of CHA2D2S-VASc score were on OACs which is consistent with AHA guidelines that emphasize stroke risk stratification and anticoagulants as an integral part of AF management. The prescription of NOAC has substantially increased while the prescription of warfarin has decreased over years.
